# Troponinemia in Patients With Diabetic Ketoacidosis Without Acute Coronary Syndrome

**DOI:** 10.7759/cureus.61064

**Published:** 2024-05-25

**Authors:** Indira Poojary, Usbah Khalid, Tumpa Patra, Junu Giri, Ammar Al Heyasat, Sayeda Basith, Saketh Palasamudram Shekar, Anand Balachandran, Akram Haggag

**Affiliations:** 1 Internal Medicine, Crestwood Medical Center, Huntsville, USA; 2 Family Medicine, Crestwood Medical Center, Huntsville, USA

**Keywords:** diabetes mellitus, diabetic ketoacidosis, acute coronary syndrome (acs), cardiac risk factors and prevention, cardiac troponin

## Abstract

Ischemic myocardial injury in a diabetes mellitus (DM) patient can be a trigger or a complication of diabetic ketoacidosis (DKA). This case series examines the phenomenon of elevated troponin levels in patients with DKA in the absence of obstructive coronary artery disease. Two out of three cases showed ST-segment elevation on electrocardiogram (EKG). Despite the absence of obstructive coronary artery disease on coronary angiography, all cases exhibited troponinemia (>79 ng/dl). These elevated troponin levels and EKG changes may pose diagnostic challenges for clinicians. Alternatively, troponinemia could be due to myocardial injury caused by acidotic stress and free fatty acid utilization along with increased myocardial oxygen demand and not obstructive coronary artery pathology in every case. However, a better understanding of the complex interplay between DKA and myocardial injury needs further research.

## Introduction

Diabetic ketoacidosis (DKA) represents a clinically significant complication that can manifest in individuals afflicted by diabetes mellitus (DM) or the presenting symptom in cases of insulin-dependent DM. Well-established triggers for DKA encompass infection, acute coronary syndrome (ACS), inadequate dietary habits, pancreatitis, drugs, gestational diabetes, and insufficient insulin levels [1]. The assessment of troponin levels serves as a specific diagnostic measure for detecting myocardial injury [2-4]. Additional indicators of myocardial ischemia comprise novel or presumed substantial ST-segment T-wave alterations, emerging left bundle branch block, the manifestation of pathological Q waves on electrocardiography, radiographic evidence of fresh loss of viable myocardial tissue, or the identification of an intracoronary thrombus through angiographic techniques [5]. A study by Manikkan in 2018 has reported instances of elevated troponin levels in DM patients with DKA without any signs of cardiac ischemia/infarctions [6,7]. An elevated level of troponin in a DM patient with DKA in the absence of ACS can pose diagnostic challenges for treating physicians when trying to determine the underlying cause of increased troponin. In this context, we present three cases of DKA where patients exhibited increased troponin levels without other indications of myocardial ischemia.

## Case presentation

Case 1

A 75-year-old African American female with a past medical history of hypertension, rheumatoid arthritis, anti-phospholipid syndrome, pyoderma gangrenosum, and pre-diabetes was admitted to the hospital after being found unresponsive by her family. Initial assessment revealed hypernatremia, elevated blood sugar, an elevated anion gap, leukocytosis, and an elevated lactic acid. Acute kidney injury (AKI) was also identified, with an elevated creatinine level. Arterial blood gas (ABG) analysis revealed metabolic acidosis.

Cardiology consultation prompted coronary angiography and left heart catheterization with left ventricular (LV) angiography, which demonstrated no significant coronary artery disease (CAD), normal LV function with an ejection fraction (EF) of 55-60%, and normal left heart hemodynamics. Her DKA was corrected with intravenous fluids, insulin, and potassium supplements. She was discharged with diabetic education and long-acting insulin and advised cardiology follow-up as an outpatient.

**Figure 1:**
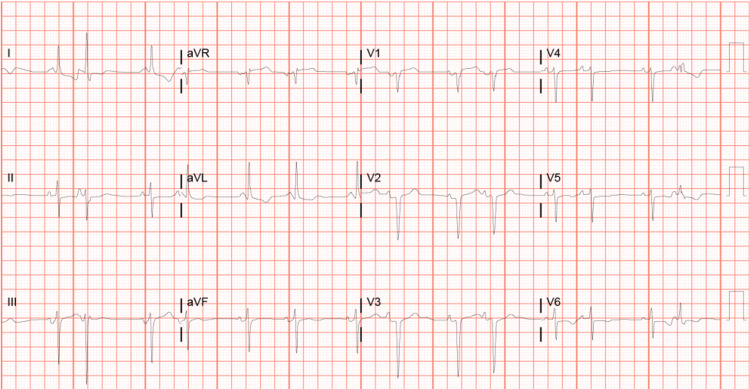
Electrocardiogram of case 1 Sinus rhythm with heart rate at 85 bpm and multiple ventricular and supraventricular premature complexes, left ventricular hypertrophy with secondary repolarization abnormality, and an old anterior infarct.

Upon admission, troponin levels were elevated, escalating sixfold within 24 hours. Electrocardiogram (EKG) findings displayed sinus rhythm with multiple ventricular and supraventricular premature complexes, LV hypertrophy (LVH) with secondary repolarization abnormality, and an old anterior infarct (Figure 1). Echocardiography revealed a normal LV size with concentric LVH and an EF ranging from 65% to 70%.

Case 2

A 39-year-old African American female with a history of non-insulin-dependent DM, non-compliant with metformin, was admitted with presenting symptoms of altered mental status, vomiting, and rapid breathing. She was having tachycardia and tachypnea on presentation, and the physical examination did not reveal any significant findings. Blood glucose on admission was elevated with an increased anion gap. The basic metabolic panel showed hyponatremia, hyperkalemia, elevated creatinine, with hypermagnesemia, and hyperphosphatemia. The lipid profile showed hypertriglyceridemia with normal cholesterol. ABG analysis showed metabolic acidosis.

Upon admission, her troponin level was notably high, increasing sixfold within 24 hours of admission. Blood cultures showed no growth. The urinalysis did not suggest any infection, and the urine toxicology screen was negative.

**Figure 2:**
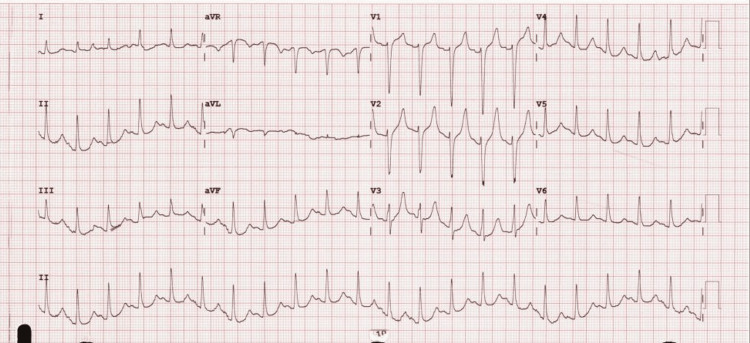
Electrocardiogram of case 2 Sinus tachycardia with heart rate at 128 bpm, ST-segment elevation in aVR, V1, and V2 and normal early repolarization pattern, and prolonged QT interval.

EKG revealed sinus tachycardia, ST-segment elevation in aVR, V1, and V2 (noncontiguous leads) and normal early repolarization pattern, and prolonged QT interval (Figure 2). Cardiac catheterization revealed no evidence of obstructive epicardial CAD, and the echocardiogram did not show any cardiac dysfunction. She was treated for DKA with intravenous fluids, insulin, and as-needed electrolyte replacement. She was discharged with diabetic education and risk factor modification and follow-up with cardiology as needed. 

Case 3

**Figure 3:**
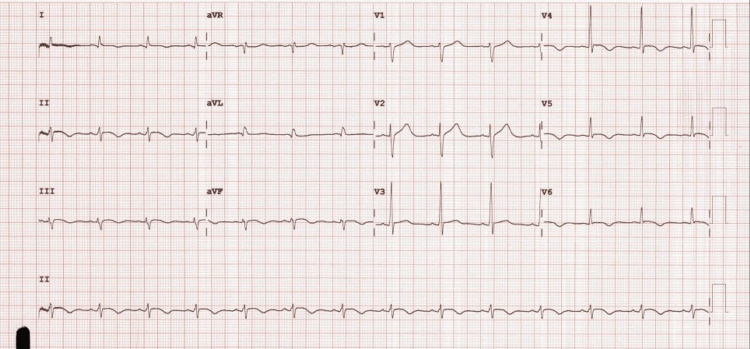
Electrocardiogram of case 3 Sinus rhythm with heart rate at 82 bpm, low voltage in extremity leads, ST-segment elevation in inferolateral leads indicating injury, and borderline QT prolongation.

A 49-year-old Caucasian male with a history of insulin-dependent DM, exhibiting noncompliance with insulin therapy, presented with symptoms of blurry vision, nausea, vomiting, and fatigue. Despite stable vital signs, he presented with mild hypothermia and signs of dehydration. Laboratory findings indicated hyponatremia, elevated anion gap, hyperglycemia, and elevated creatinine. Initial cardiac markers revealed normal troponin, but troponin levels subsequently elevated exponentially within 24 hours. EKG showed ST-segment elevation in inferolateral leads consistent with acute myocardial infarction (MI) (Figure 3).

The patient underwent immediate cardiac catheterization, which revealed no evidence of CAD. However, moderate to severe LV systolic dysfunction was observed, accompanied by a dilated left ventricle and an EF of 30%. Additionally, inferoapical and apical akinesis was noted, consistent with Takotsubo cardiomyopathy. The patient was given intravenous fluids, insulin, and electrolyte replacement for DKA management. He was discharged on carvedilol, spironolactone, and lisinopril and advised regular follow-up.

Table 1 depicts the demographic information and laboratory investigations at admission of cases 1, 2, and 3.

**Table 1:** Demographic information and laboratory investigations at admission *These investigations were not done for case 3 LDL: low-density lipoprotein; HDL: high-density lipoprotein; pCO2: partial pressure of carbon dioxide; PO2: partial pressure of oxygen; HCO3: bicarbonate; WBC: white blood cell

Demographics	Case 1	Case 2	Case 3	-
Age	75	39	49	-
Gender	Female	Female	Male	-
	Observed value			Reference range
Electrolytes				
Sodium	165	132	124	136-145 mEq/l
Potassium	3.7	5.5	4.9	3.5-5 mEq/l
Chloride	133	99	91	98-110 mEq/l
Anion gap	23.7	36.5	29.9	6-14.3 mEq/l
Serum glucose	434	881	753	70-100 mg/dl
Creatinine	2.1	2.5	2.0	0.6-1.3 mg/dl
Phosphate	3.8	9.6	1.1	2.5-4.9 mEq/l
Magnesium	3.2	2.8	1.5	1.8-2.4 mEq/l
Lactic acid	2.1	3.0	1.6	0.4-2 mEq/l
Lipid profile*				
Cholesterol	178	139	-	<200 mg/dl
Triglyceride	269	240	-	<150 mg/dl
LDL	75	58	-	>40 mg/dl
HDL	49	33	-	<100 mg/dl
Arterial blood gas*				
pH	7.21	7.25	-	7.35-7.45
pCO2	21.5	13.7	-	35-45
PO2	91	109	-	80-105
HCO3	8.6	6.0	-	22-26
Cardiac enzymes				
Troponin (at admission)	1076	989.6	6889.3	0-79 ng/l
Troponin (in 24 hours)	5817	6675.3	>25000	0-79 ng/l
Blood counts				
WBC	16,300	26,200	51,000	4000-11000 cells/dl

## Discussion

Myocardial ischemia/MI as a potential trigger and serious complication of DKA is pertinent, particularly in light of elevated troponin levels serving as a diagnostic indicator of MI. Notably, DM represents a significant risk factor for obstructive CAD, which could lead to ACS. However, recent studies have illustrated an elevation in troponin levels among DKA patients without a history of obstructive CAD [6-8]. In our case series, we present three cases with a sixfold increase in troponin levels in the absence of obstructive coronary pathology.

Eubanks et al., in a retrospective study, proposed that the acidotic environment caused by DKA increases intracellular calcium, leading to proteolysis, myocardial stunning, damage, and a rise in troponin levels. The study also explained that the increase in counter-regulatory hormones during DKA could lead to heightened myocardial oxygen demand and troponinemia [9]. Umpierrez et al. discussed that in an insulin-deficient state, the myocardium's utilization of free fatty acids, which are toxic to the myocardium, can result in myocardial damage and troponinemia. This state can also increase free radical damage to the myocardium [10]. Several studies stated that hyperkalemia can present with ST-segment elevation and pseudo-infarct pattern with resolution after the hyperkalemia is resolved [11-13] which was seen in case 2. Another study suggested that elevated troponin levels are associated with a very high risk of future cardiac events [7]. One of our cases had evidence of Takotsubo cardiomyopathy, and there are case reports of similar findings in DKA patients [14,15]. Intracellular increase in calcium with proteolysis due to acidemia, free fatty acid-mediated damage to the myocytes, increase in counter-regulatory hormone causing increased myocardial oxygen demand, and pro-inflammatory cytokines increasing free radicles that inhibit the contractile proteins and cause myocardial stunning may be some of the mechanisms by which cardiomyopathy and troponinemia are seen in DKA [16,17]. Additionally, a retrospective study by Al-Mallah et al. assessed the prognostic importance of troponin I elevation among patients presenting with DKA without ACS. They found that this group of patients had a significant risk of major adverse coronary event rate at two years follow-up [7]. The findings from these studies indicate the lack of evidence and the need for further studies to stratify the risk and long-term outcomes among DKA patients with elevated troponin in the absence of ACS. 

In the absence of occlusive myocardial infarction (OMI), microinfarctions, and small vessel disease, troponinemia without obstructive CAD should be considered as differential diagnosis in DKA patients. Hence, it's important for clinicians to carefully consider the clinical presentation, diagnostic results, and patient history when determining the most likely diagnosis. Our study limitation includes lack of ability to generalize due to a small number of cases from one hospital.

## Conclusions

This case series emphasizes instances of high troponin levels without ACS in patients with DKA. Few case reports have presented comparable results, leading to the formulation of various hypotheses that require additional verifications. Considering these findings together, we advocate more extensive research, including larger participant groups and extended patient monitoring, to determine whether elevated troponin in DKA patients is linked to adverse long-term cardiac outcomes.
